# GPA Peptide-Induced Nur77 Localization at Mitochondria Inhibits Inflammation and Oxidative Stress through Activating Autophagy in the Intestine

**DOI:** 10.1155/2020/4964202

**Published:** 2020-08-20

**Authors:** Zhao Deng, Qi Liu, Miaomiao Wang, Hong-Kui Wei, Jian Peng

**Affiliations:** ^1^Department of Animal Nutrition and Feed Science, College of Animal Science and Technology, Huazhong Agricultural University, 430070 Wuhan, China; ^2^The Cooperative Innovation Center for Sustainable Pig Production, Wuhan, 430070 Hubei, China

## Abstract

Inflammatory bowel disease (IBD), including Crohn's disease (CD) and ulcerative colitis (UC), is a chronic inflammatory disease affecting the colon, and its incidence is rising worldwide. Nur77, belongs to the NR4A subfamily of nuclear hormone receptors, plays a critical role in controlling the pathology of colitis. The aim of this study is to investigate the protection effect and mechanism of Gly-Pro-Ala (GPA) peptide, isolated from fish skin gelatin hydrolysate, in a mouse model of dextran sulfate sodium- (DSS-) induced colitis and intestinal epithelial cells (IECs) stimulated by lipopolysaccharide (LPS). In vivo, GPA treatment alleviates DSS-induced weight loss, disease activity index (DAI) increase, colon length shortening, and colonic pathological damage. Production of proinflammatory cytokines, ROS, and MDA is significantly decreased by GPA treatment. In vitro, GPA significantly inhibits proinflammatory cytokine production, cytotoxicity, ROS, and MDA in IECs. Furthermore, GPA induces autophagy to suppress inflammation and oxidative stress. GPA promotes Nur77 translocation from the nucleus to mitochondria where it facilitates Nur77 interaction with TRAF6 and p62, leading to the induction of autophagy. In addition, GPA contributed to the maintenance of tight junction architecture in vivo and in vitro. Taken together, GPA, as a Nur77 modulator, could exert anti-inflammatory and antioxidant effects by inducing autophagy in IECs, suggesting that GPA may be promising for the prevention of colitis.

## 1. Introduction

Inflammatory bowel disease (IBD), including ulcerative colitis (UC) and Crohn's disease (CD), is a chronic and relapsing inflammatory condition of the gastrointestinal tract, resulting in weight loss, diarrhea, rectal bleeding, and abdominal pain [1–3]. IBD has become a globally widespread disease in the past decade, affecting millions of people worldwide [2–4]. Although the etiology and pathogenesis of IBD are complicated and remain unclear, environmental, genetic, and lifestyle factors, immune system, intestinal microflora, and intestinal epithelial barrier have been found to be involved in the pathology of IBD [5–7].

Under IBD, intestinal microbiota in the lumen are altered, the intestinal epithelial barrier is disrupted, and then a lot of proinflammatory cytokines and ROS are produced, leading to amplify immune response and a cycle of uncontrolled inflammation [8, 9].

The inflammation and oxidative stress are considered to play a crucial role in the pathogenesis of colitis, where the intestinal epithelial cells (IECs) are first line of defense against infection in the gut, serving as immune surveillance [10]. Hence, the strategies for treating IBD have been focused on blocking inflammation and oxidative stress [8, 9].

Nur77 (also called TR3, NGFIB, or NR4A1), a member of the NR4A family of nuclear receptors, plays a crucial role in controlling inflammatory diseases, including colitis, diabetes, sepsis, and pulmonary hypertension [11–13]. The level of Nur77 is significantly decreased in colon tissues from patients with IBD and mouse model of colitis, whereas the Nur77 deficiency exacerbates colitis in mice [14]. Recently, several studies have focused on its potent anti-inflammatory effect in inflammatory diseases [14–17]. Mitochondrial function emerges as a crucial role in coordinating cellular metabolism, immunity, stress responses, inflammatory processes, and apoptosis, and it develops a gatekeeper in IEC homeostasis [18]. Thus, mitochondria emerge as a crucial cellular checkpoint at the edge of tissue homeostasis and pathology, becoming a potential target of IBD [19]. Previous research has showed that Nur77 could translocate from the nucleus to mitochondria to induce autophagy [20, 21]. Whether Nur77 participates in modulating mitochondrial function and mediating inflammatory signaling in IBD remains unknown. Therefore, it is our concern whether controlling the mitochondrial function in IECs through Nur77 can be an effective way to alleviate inflammatory response and maintain intestinal homeostasis.

Numerous studies have reported that some nutrients, food components, and bioactive peptides exert anti-inflammatory effects in inflammatory diseases [22, 23]. Here, we report our discovery of Gly-Pro-Ala (GPA) peptide, isolated from fish skin gelatin hydrolysate [24, 25], could possess anti-inflammatory and antioxidant effects in vitro and in vivo. Our results demonstrate that GPA, as a Nur77 modulator, induces Nur77 mitochondrial translocation and subsequently inhibits inflammation and oxidative stress through inducing autophagy.

## 2. Materials and Methods

### 2.1. Reagents and Antibodies

Synthetic GPA peptide was purchased from Top Peptide Biotechnology Co., Ltd. (Shanghai, China). Dextran sulfate sodium (DSS, molecular weight of 36–50 kDa) was purchased from MP Biomedicals (Irvine, CA, USA). LPS (L6230) was purchased from Sigma-Aldrich (St. Louis, MO, USA). Murine TNF-*α* recombinant protein (315-01A-20) was purchased from PeproTech (USA). Chloroquine diphosphate (HY-17589) was purchased from MedChemExpress (St. Louis, MO, USA). 3-MA (T1879) and Protease Inhibitor Cocktail (C0001) were purchased from Target Mol (Topscience, Shanghai, China). Cell lysis buffer for Western analysis (P0013), phenylmethanesulfonyl fluoride (PMSF) (ST505), and LDH detection kits (C0017) were purchased from Beyotime (Shanghai, China). DAPI (D4054) was purchased from US Everbright® Inc. (Suzhou, China). The antibodies against I*κ*B*α* (A1187), p62 (A7758), LC3B (A11282), and *β*-actin (AC026) were purchased from Abclonal (Wuhan, China). The antibodies against ZO-1 (AF5145) and occludin (DF7504) were purchased from Affinity (Cincinnati, USA). The antibody against TRAF6 (66498-1) was brought from Proteintech (Wuhan, China). The antibodies against IL-6 (GB11117) and GAPDH (GB1102) were purchased from Servicebio (Wuhan, China).

### 2.2. Cell Culture

MODE-K cells, an intestinal epithelial cell line derived from C3H/HeJ mice [26], were purchased from BeNa Culture Collection (Beijing, China). The MODE-K cell culture model served as a system to study gut epithelial cells' bidirectional signaling responses and to understand the modulating effects of dietary bioactive components [27]. MODE-K cells were cultured in RIPA 1640 (Gibco, San Diego, CA, USA) containing 10% fetal bovine serum (FBS, Gibco, San Diego, CA, USA) and 1% penicillin/streptomycin at 37°C under a 5% CO_2_ atmosphere.

### 2.3. Animals

Male C57BL/6 mice (5 weeks old) obtained from the Animal Experiment Center at Huazhong Agricultural University (Wuhan, China) were used for the current study. Mice were housed under specific pathogen-free conditions in an air-conditioned room at 23 ± 2°C. Food and water were supplied ad libitum. All animal experimental protocols were approved by the Institutional Animal Care and Use Committee of Huazhong Agricultural University. All efforts were made to minimize animal suffering and to reduce the number of animals used.

### 2.4. Establishment of DSS-Induced Mouse Colitis Model and Treatment

Acute colitis was induced by feeding mice with 3% (*w*/*v*) DSS, which was dissolved in drinking water, continuously for 7 days. The experiment was randomly divided into three groups: control group, DSS group, and GPA (100 mg/kg) + DSS group. To assess the chemoprevention effect of GPA on DSS-induced acute colitis in C57BL/6 mice, the mice were treated with 3% DSS in their drinking water for 7 days to induce acute colitis. GPA (100 mg/kg) was administered for 7 days before and during DSS treatment via oral gavage once per day. After 14 days, mice were humanely euthanized, and the colons were excised, measured, and sectioned for further analysis [28, 29].

### 2.5. Evaluation of Colitis Severity

We evaluated the colitis severity on the basis of body weight, colon length, and the macroscopic and microscopic observations of the stool and colon, respectively. The disease activity index (DAI) score was determined by the method employed in previous studies, using five grades of weight change (0, no weight loss or gain; 1, 1-5% loss; 2, 5-10% loss; 3, 10-20% loss; and 4, more than 20% loss), stool consistency (0, normal; 1, mild loose; 2, loose; 3, mild diarrhea; and 4, diarrhea), and stool bleeding (0, negative; 1, light bleeding; 2, mild bleeding; 3, severe bleeding; and 4, complete bleeding). Colon sections were prepared and stained with hematoxylin and eosin (H&E) according to standard protocols. Histological scoring was performed using a previously described method [30, 31].

### 2.6. Cytotoxicity Assay

Cells were treated as indicated. Cytotoxicity was determined by measuring the release of lactate dehydrogenase (LDH) Beyotime (Shanghai, China), following the manufacturer's instructions. LDH level presented a relative content, corrected with a blank control (without cells); the percentage of LDH release was calculated as(experimental LDH − spontaneous LDH)/(maximum LDH release − spontaneous LDH) × 100, as previously described [32].

### 2.7. RNA Isolation and Quantitative Real-Time PCR

Total RNA was extracted using the TRIzol reagent (Invitrogen) and transcribed into cDNA using a First-Strand cDNA Synthesis Kit (TOYOBO, Japan). Quantitation of the mRNA level by quantitative real-time (qPCR) was performed on a real-time PCR system (Bio-Rad, Richmond, CA, USA) using iTaq Universal SYBR Green Supermix (Bio-Rad, Richmond, CA, USA). The mean of the triplicate cycle thresholds (Ct) of the target gene was normalized to the mean of triplicate Ct of the reference *β*-actin and GAPDH gene using the formula “2^-*ΔΔ*Ct^,” which yielded relative gene expression level values. The primers used are listed in Supplementary Table 1.

### 2.8. RNA Interference and Transfection

Synthetic siRNA oligonucleotides specific for regions in the mouse Nur77, TRAF6, and p62 mRNA were designed and synthesized by GenePharma (Shanghai, China). The primers used are listed in Supplementary Table 2. Cells were transfected at 70%–80% confluence with siRNA duplexes using Lipofectamine RNAiMAX (Invitrogen, Carlsbad, CA, USA) in accordance with the manufacturer's instructions.

### 2.9. Immunoblot Analysis: Immunoprecipitation (IP)

The cells were extracted with protein lysis buffer (Beyotime, China) supplemented with protease inhibitor cocktail. Protein concentration was determined using the BCA Kit (Beyotime, China). Proteins (25–35 *μ*g) were separated on a 10% polyacrylamide precast SDS gel (Bio-Rad, Richmond, CA, USA) followed by blotting on PVDF membranes (Millipore Billerica, MA, USA). For IP experiments, cells were lysed in IP buffer (Beyotime, China) and incubated with IP-grade antibodies followed by the pull-down with protein A/G beads (161-4023) (Bio-Rad, Richmond, CA, USA) for subsequent immunoblot analyses.

For pull-down assay, MODE-K cell lysates were collected and centrifuged at 8000 × *g*. The supernatant was transferred to another tube, and the cell debris was thoroughly discarded. Prewashed streptavidin beads were added into the supernatant, allowing 2 h preincubation with motion at 4°C to remove unspecific binding proteins. Purified mouse recombinant Nur77-LBD proteins were dissolved in lysis buffer. The lysates or recombinant proteins were incubated with compounds, followed by incubation with indicated doses of biotin-GPA for 6 h. Beads were washed with IP buffer for three times and boiled in SDS buffer.

### 2.10. Immunofluorescence and Confocal Imaging

Cells were washed twice with PBS and fixed in 4% paraformaldehyde for 30 min at room temperature. 4,6-Diamidino-2-phenylindole (DAPI) (Molecular Probes) was used to label DNA. Confocal imaging was performed using a confocal laser scanning microscope (Carl Zeiss, Germany) equipped with an incubation chamber and a motorized table. Mitochondria were marked by MitoTracker (Red) (1 : 10000 dilution) for 30 min before fixed by 4% buffered formalin/PBS.

### 2.11. ELISA

Supernatants from cell culture were diluted for MCP-1 detection (DAKEWE, Beijing, China). TNF-*α* in serum was analyzed directly using an ELISA kit (NEOBIOSCIENCE, Shenzhen, China) according to the manufacturer's instructions. For TNF-*α* concentrations in mouse colon tissues, tissues were homogenized in five volumes of ice-cold PBS containing 1 mM PMSF and 10 mM phosphatase inhibitor (Servicebio) and centrifuged at 12,000 × *g* for 10 min at 4°C. Protein concentration was measured by BCA Kit (Beyotime, China), and TNF-*α* was detected by ELISA kit.

### 2.12. Immunohistochemistry (IHC)

Immunohistochemical stains against ZO-1 and occludin were detected using IHC kit (MaiXin, China). Briefly, paraffin-embedded slides were deparaffinized, rehydrated, and washed in 1% PBS. Afterwards, they were incubated with 3% hydrogen peroxide and blocked with 10% goat serum for 1 h at 37°C. Then, slides were treated with primary antibodies (1 : 100) overnight at 4°C. Biotinylated secondary anti-rabbit antibodies were added and incubated at room temperature for 1 h. Streptavidin-HRP was added, and after 40 min, the sections were stained with a diaminobenzidine as a chromogen and counterstained with hematoxylin. Images at 200x magnification were examined with a microscope (Olympus, Japan).

### 2.13. Determination of Transepithelial Electrical Resistance

Caco-2 cells (1 × 105 cells/well) were seeded in Transwell inserts (membrane area 0.33 cm^2^, pore size 0.4 *μ*m) placed in 24-well plates. Transepithelial electrical resistance (TEER) was monitored daily using an EVOM voltohmmeter with STX2 electrodes (World Precision Instruments). When the resistance approached 1000 *Ω* cm^2^, cells were pretreated with or without GPA for 6 h followed by treatment with LPS. TEER was measured hourly during 6 h LPS treatment.

### 2.14. Measurement of ROS and MDA Production

The fluorescent probe DCFH_2_-DA was used to detect the formation of intracellular ROS. Briefly, MODE-K cells (5 × 10^4^ cells/well) were seeded in a 24-well plate to reach 80% confluence. After GPA pretreatment for 6 h followed by LPS incubation for 3 h, the cells were incubated with 10 *μ*M DCFH_2_-DA at 37°C for 30 min. Finally, cells were washed with PBS for three times, and the fluorescence was quantified on a FACSCalibur cytometry system (BD Biosciences) with excitation at 488 nm and emission at 530 nm. The results were expressed as percent of control values [24, 25, 31, 33].

MDA was analyzed directly using kits according to the manufacturer's instructions, purchased from Nanjing Jiancheng Bioengineering Institute (Nanjing, China). After treatment, colon samples were excised and homogenized immediately at 4°C, and cells were broken by ultrasonic breaker on the ice. Protein concentration was determined quantitatively with a BCA protein assay kit. MDA activity in colonic tissues, serum, and cells was measured by chemical chromatometry using a relevant assay kit (Nanjing Jiancheng Institute of Bioengineering, Nanjing, China), as previously described [34].

### 2.15. Mitochondria Isolation Technique

Mitochondria isolation kit (C3601) was purchased from Nanjing Jiancheng Institute of Bioengineering (Nanjing, China). Wash the cells with PBS and then digest the cells with 100-200 g/min of trypsin-EDTA solution. Next, centrifuge it at room temperature for 5 or 10 minutes to collect cells. Cell precipitation is gently suspended with ice bath precooled PBS. A small number of cells were taken for counting, and the rest of cells are centrifuged at 600 g/min and 4°C for 5 minutes. Abandon supernatant. Adding 1-2.5 ml mitochondrial isolation reagent or mitochondrial isolation reagent added with PMSF before use to 20-50 million cells, gently suspend the cells and leave on ice bath for 10-15 minutes. Transfer the cell suspension to a glass homogenizer of appropriate size and homogenate for about 10-30 times. Centrifuge the cell homogenate at 600 g/min and 4°C for 10 minutes. Carefully transfer the supernatant to another centrifugal tube and centrifuge at 11000 g/min and 4°C for 10 minutes. Remove the supernatant carefully. Precipitation is the isolated mitochondria of cells, next detecting protecting by western blot.

### 2.16. Statistical Analysis

Data are presented as the mean ± standard error of the mean. Differences between group means were determined by one-way ANOVA using SAS 8.0 software. The Tukey post hoc multiple comparison test was performed to compare significant variations. Differences were considered significant at *p* < 0.05.

## 3. Results

### 3.1. GPA Attenuates DSS-Induced Colitis In Vivo

**Figure 1:**
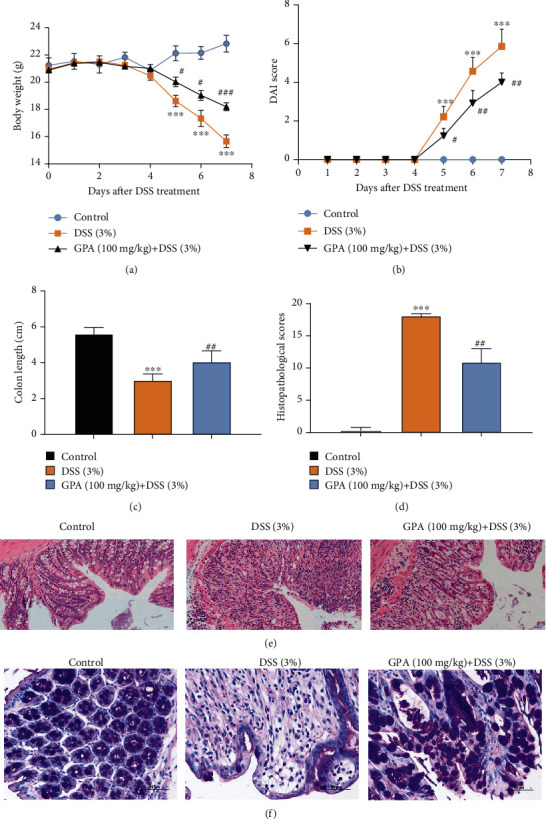
GPA attenuates DSS-induced colitis in vivo. Mice were treated with 3% DSS in their drinking water for 7 days to induce acute colitis. GPA (100 mg/kg) was administered for 7 days before and during DSS treatment via oral gavage once per day. Mice (*n* = 10/group) were sacrificed at day 14. Weight of mice during the experiment (a). Disease activity index (DAI) during the disease process (b). The lengths of the colons from each group of mice were measured (c). The colons from each experimental group were processed for histological evaluation by H&E staining; histopathological scores of each group were determined (d, e). Globe cells were determined by periodic acid-Schiff (PAS) staining (f). The results are expressed as the mean ± SD (*n* = 10/group). ^∗^*p* < 0.05, ^∗∗^*p* < 0.01, and ^∗∗∗^*p* < 0.001 vs. the control group; ^#^*p* < 0.05, ^##^*p* < 0.01, and ^###^*p* < 0.001 vs. the DSS-treated group.

To evaluate the protective effect of GPA peptide on inflammatory diseases, we established a model of DSS-induced mouse colitis by feeding C57BL/6 mice with drinking water containing 3% DSS for 7 days. The mice treated with only DSS had a significant decrease in body weight and DAI scores compared with the control group. The supplementation of GPA at a dose of 100 mg/kg body weight significantly attenuated the DSS-induced weight loss and DAI scores compared to DSS administration alone (Figures 1(a) and 1(b)). In addition, DSS typically caused colonic shortening, whereas this change was significantly improved in the GPA groups (Figure 1(c)). The severity of colonic inflammation was further evaluated by histopathological analysis. The DSS group exhibited distortion of crypts, loss of goblet cells, and severe mucosal damage. However, administration of GPA to DSS-induced IBD mice clearly improved the pathological changes (Figures 1(d)–1(f)). Meanwhile, we evaluated GPA alone treatment on mice; the results showed that GPA alone treatment could not affect mouse weight change, DAI score, and colon shortening (Figure S1A-C). These results demonstrated that GPA could significantly reduce the level of colitis induced by DSS.

### 3.2. GPA Reduces Proinflammatory Cytokine Secretion and Oxidative Stress In Vivo

**Figure 2:**
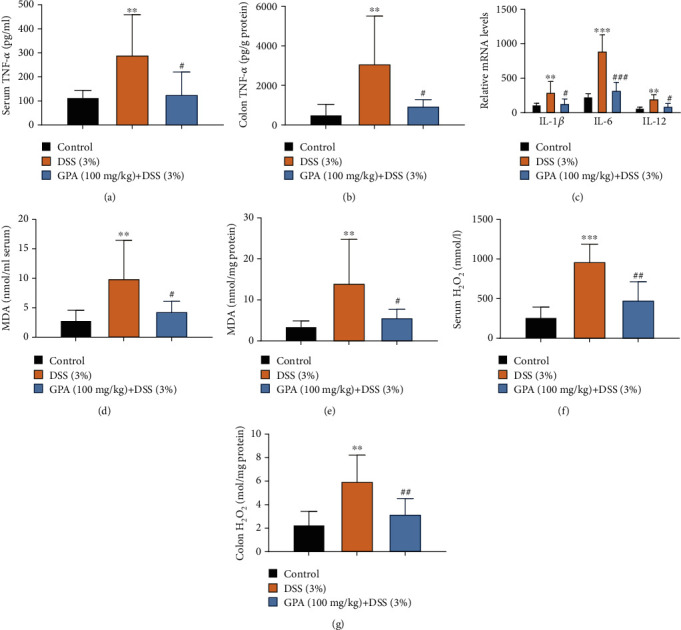
GPA reduces proinflammatory cytokine secretion and oxidative stress in vivo. The production of inflammation-related cytokine tumor necrosis factor *α* (TNF-*α*) in serum was measured by ELISA (a). The production of inflammation-related cytokine TNF-*α* in colon tissues by ELISA (b). IL-1*β*, IL-6, and IL-12 in mouse colon tissues were detected by qPCR (c). MDA content in serum and colon tissues were measured (d, e). H_2_O_2_ content in serum and colon tissues were measured (f, g). The results are expressed as the mean ± SD (*n* = 10/group). ^∗^*p* < 0.05, ^∗∗^*p* < 0.01, and ^∗∗∗^*p* < 0.001 vs. the control group; ^#^*p* < 0.05, ^##^*p* < 0.01, and ^###^*p* < 0.001 vs. the DSS-treated group.

The anticolitis effects of GPA were further confirmed by detecting proinflammatory cytokines. We detected the concentration of tumor necrosis factor-*α* (TNF-*α*) in serum and colon tissue by ELISA, and the results showed that GPA significantly inhibited the expression of TNF-*α* (Figures 2(a) and 2(b)). Next, we evaluated other cytokines in colon tissues by qPCR, i.e., IL-1, IL-6, and IL-12; it showed that GPA significantly inhibited proinflammatory cytokine production (Figure 2(c)).

Intestinal oxidative stress has been associated with the initiation and propagation of chronic intestinal pathologies such as IBD [35]. Then, we evaluated the state of oxidative stress in DSS-induced mouse colitis. MDA (malondialdehyde), one of the main end products of lipid peroxidation reaction, is an important marker of oxidative stress in acute colitis [34]. Then, we detected the level of MDA in serum and colon tissues. The results showed DSS increased level of MDA, and GPA treatment significantly reduced MDA in serum and colon tissues (Figures 2(d) and 2(e)). Next, we detected H_2_O_2_, as one of ROS [36, 37], in serum and colon tissues; the results showed that GPA treatment significantly reduced H_2_O_2_ in serum and colon tissues (Figures 2(f) and 2(g)). These results demonstrated that GPA could significantly inhibit inflammation and oxidative stress in DSS-induced colitis.

### 3.3. GPA Inhibits Inflammation and Oxidative Stress in IECs

**Figure 3:**
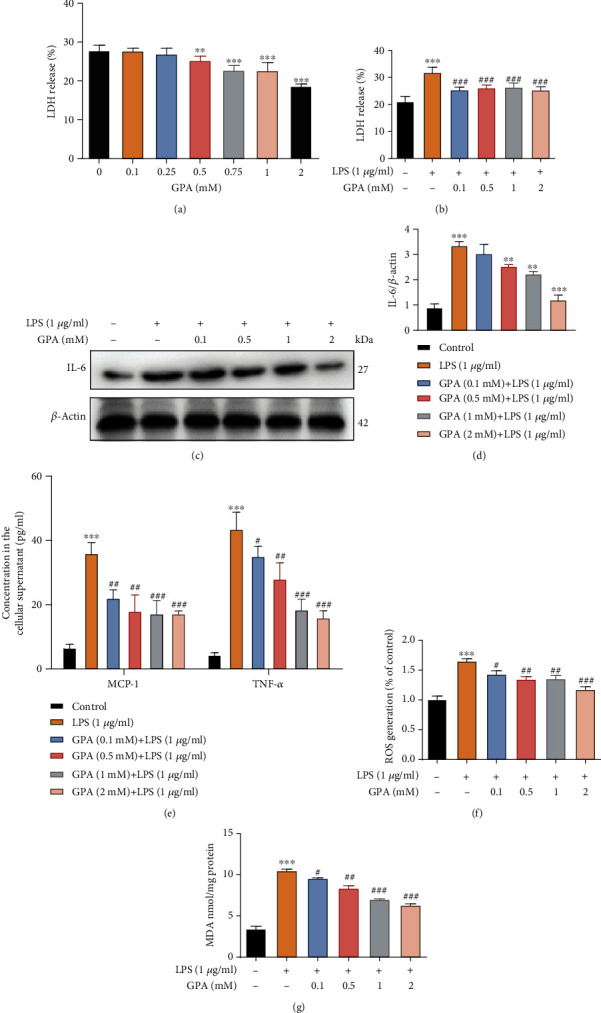
GPA inhibits inflammation and oxidative stress in IECs. MODE-K cells were treated with different GPA concentrations for 6 h, and the cytotoxicity was detected by LDH assay (a). MODE-K cells pretreated with different concentrations of GPA for 6 h were exposed to LPS (1 *μ*g/ml) for 3 h, and the cytotoxicity was detected by LDH assay (b). The level of IL-6 was detected by western blot (c, d). MCP-1 and TNF-*α* levels were detected by ELISA (e). The intracellular ROS levels were measured by DCF fluorescence microscopy (f). And the content of MDA was determined (g). The results are expressed as the mean ± SD, three independent experiments. ^∗^*p* < 0.05, ^∗∗^*p* < 0.01, and ^∗∗∗^*p* < 0.001 vs. the control group; ^#^*p* < 0.05, ^##^*p* < 0.01, and ^###^*p* < 0.001 vs. the LPS-treated group.

Next, we evaluated the protective effect of GPA on inflammation and oxidative stress in vitro. Then, we examined the potential anti-inflammatory and antioxidant properties of GPA, using an in vitro model of LPS or TNF-*α*-induced inflammation in IECs. GPA treatment significantly reduced LDH released from MODE-K cells in a dose-dependent manner (Figure 3(a)), without affecting cell viability (Figure S2A). And GPA also significantly decreased LDH secretion induced by LPS in MODE-K cells (Figure 3(b)).

Treatment with GPA significantly reduced the levels of interleukin-6 (IL-6), as well as monocyte chemoattractant protein-1 (MCP-1) and TNF-*α*, in MODE-K cells compared to cells treated with LPS alone (Figures 3(c), 3(d), and 3(e)). Then, we confirmed the results by qPCR; GPA suppressed proinflammatory cytokine production in MODE-K cells (Figure S2B). Likewise, we also found that GPA inhibited TNF-*α*-induced LDH released MCP-1 and IL-6 production in a dose-dependent manner in MODE-K cells (Fig. S3AB). For oxidative stress, we detected level of ROS and MDA in IECs; the results showed that GPA treatment significantly inhibited ROS and MDA production induced by LPS (Figures 3(f) and 3(g)). The results indicated that GPA could reduce proinflammatory cytokine production and oxidative stress in IECs.

### 3.4. GPA Contributed to the Maintenance of TJ Architecture In Vivo and In Vitro

**Figure 4:**
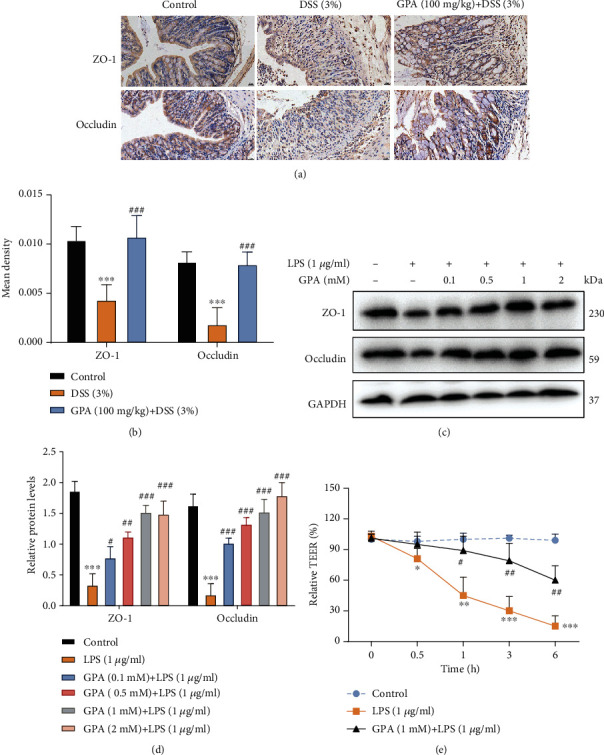
GPA contributed to the maintenance of TJ architecture in vivo and in vitro. The levels of ZO-1 and occludin were analyzed by IHC in mouse colon tissues (a, b). The results are expressed as the mean ± SD (*n* = 6/group). ^∗^*p* < 0.05 vs. the control group; ^#^*p* < 0.05 vs. the DSS-treated group. IPEC-J2 cells pretreated with different concentrations of GPA for 6 h were exposed to LPS (1 *μ*g/ml) for 3 h. ZO-1 and occludin protein levels were analyzed by western blotting in IPEC-J2 cells (c, d). In vitro IPEC-J2 cell permeability was monitored using the transepithelial electrical resistance (TEER) assay (f). The results are expressed as mean ± SD, three independent experiments. ^∗^*p* < 0.05, ^∗∗^*p* < 0.01, and ^∗∗∗^*p* < 0.001 vs. the control group; ^#^*p* < 0.05, ^##^*p* < 0.01, and ^###^*p* < 0.001 vs. the LPS-treated group.

Epithelial tight junction (TJ) proteins are an especially important aspect of the mechanical barrier, preventing harmful substances from breaching the mucosa, maintaining cellular integrity and permeability, and ensuring a relatively stable internal environment [38, 39]. ZO-1 and occludin are important epithelial TJ proteins. Thus, we detected the effect of GPA on epithelial TJ proteins (ZO-1 and occludin) in colon tissues; ZO-1 and occludin-positive signals were obviously decreased after DSS administration by IHC compared with the control group, while these changes were significantly increased through pretreatment with GPA in colon tissues (Figures 4(a) and 4(b)).

Pretreatment with GPA significantly alleviated the loss of ZO-1 and occluding caused by LPS in IECs (Figures 4(c) and 4(d)). TEER exhibited a marked reduction in a time-dependent manner after LPS treatment, while it was alleviated by pretreatment with GPA in IECs (Figure 4(e)). These findings suggested the importance of GPA for restoring the integrity of the TJ networks.

### 3.5. GPA Induces Autophagy to Suppress Inflammation and Oxidative Stress

**Figure 5:**
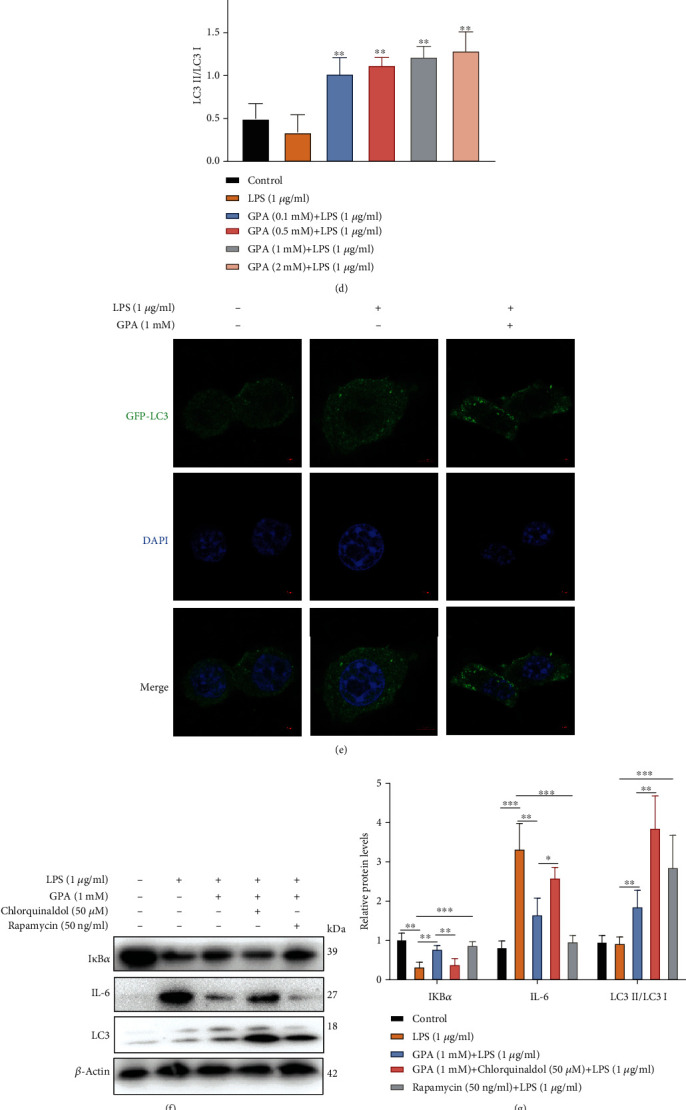
GPA induces autophagy to suppress inflammation and oxidative stress. LC3 protein levels were analyzed by IHC in mouse colon tissues (a, b). The results are expressed as the mean ± SD (*n* = 6/group). ^∗^*p* < 0.05 vs. the control group; ^#^*p* < 0.05 vs. the DSS-treated group. MODE-K cells pretreated with different concentrations of GPA for 6 h were exposed to LPS (1 *μ*g/ml) for 3 h. The protein level of LC3 was analyzed by western blot (c, d). Representative immunofluorescence images show the formation of GFP-LC3 punctate in MODE-K cells after treatment with GPA and LPS. GFP-LC3 (green) and DAPI (blue). Scale bar, 5 *μ*m (e). MODE-K cells pretreated with chlorquinaldol or rapamycin (50 ng/ml) for 12 h were exposed to GPA (1 mM) for 6 h, followed by incubation with LPS for 3 h, and then, the levels of LC3, IL-6, and IKB*α* were detected by western blot (f, g). The intracellular ROS levels were measured by DCF fluorescence microscopy (h). The results are expressed as the mean ± SD, three independent experiments. ^∗^*p* < 0.05, ^∗∗^*p* < 0.01, and ^∗∗∗^*p* < 0.001 vs. the control group; ^#^*p* < 0.05, ^##^*p* < 0.01, and ^###^*p* < 0.001 vs. the LPS-treated group.

Recent research has revealed a crucial role of the autophagy pathway in regulating inflammation and oxidative stress [40–44]. We next determined whether the anti-inflammatory and antioxidant effect of GPA could be attributed to its induction of autophagy. To this end, we measured the level of LC3, a marker of autophagy, in the mouse colon tissues by IHC and immunofluorescence. The results showed that GPA activated autophagy in DSS-induced mouse colitis (Figures 5(a) and 5(b) and Figure S4A). To further examine whether GPA activates autophagy in IECs, western blot was performed, revealing a strong induction of LC3 upon treatment of MODE-K cells with GPA (Figures 5(c) and 5(d)). The autophagic effect of GPA was also demonstrated by the fact that it induced the formation of punctate green fluorescent protein-LC3 (GFP-LC3), a hallmark of autophagy induction (Figure 5(e)). We also found that GPA induced autophagy in TNF-*α*-induced inflammation model (Figure S4B). Furthermore, GPA significantly increased the mitochondrial translocation of LC3 (Figure S4C), indicating that mitophagy was activated after GPA treatment in IECs.

An inhibitor and activator of autophagy were further used to test whether GPA inhibits inflammation and oxidative stress through activating autophagy. The results showed that treatment with chlorquinaldol (an inhibitor of autophagy) blocked the effects of GPA on IL-6, I*κ*B*α*, and ROS production, while rapamycin (an activator of autophagy) obviously decreased inflammation and ROS production compared to treatment with LPS alone (Figures 5(f)–5(h)). These results addressed that GPA could exert anti-inflammatory and antioxidant effects on intestinal inflammation through inducing autophagy.

### 3.6. GPA Promotes Nur77 to Colocalization at Mitochondria to Induce Autophagy

**Figure 6:**
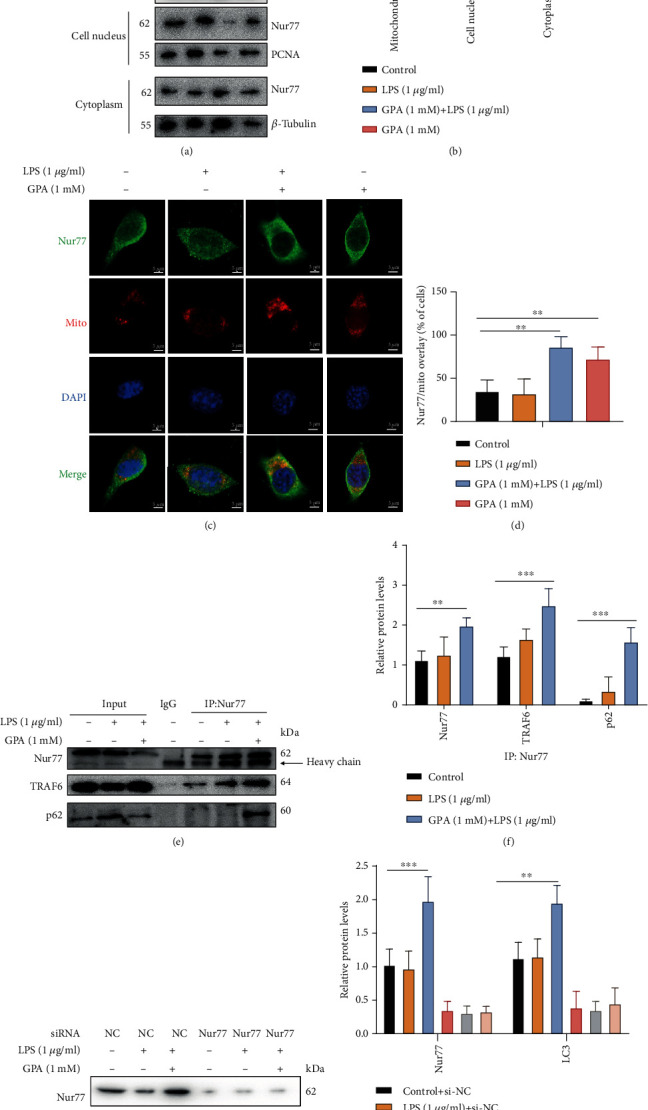
GPA promotes Nur77 to colocalization at mitochondria to induce autophagy. MODE-K cells were stimulated with GPA (1 mM) for 6 h, followed by LPS (1 *μ*g/ml) for 3 h. Mitochondria were separated by Cell Mitochondria Isolation Kit, and the level of Nur77 in the mitochondria, cell nucleus, and cytoplasm was detected by western blot (a, b). Representative images show colocalization of endogenous Nur77 with mitochondria in MODE-K cells after treatment with GPA (1 mM) and/or LPS (1 *μ*g/ml). Nur77 (green), mitochondria (red), and DAPI (blue). Scale bar, 20 *μ*m (c, d). IP and immunoblot analysis of the interaction of endogenous Nur77 with TRAF6 and p62 (e, f). MODE-K cells were transfected with control or Nur77siRNA and further stimulated with GPA (1 mM) for 6 h, followed by LPS (1 *μ*g/ml) for 3 h; levels of Nur77 and LC3 in cell lysate were analyzed by western blot (g, h), three independent experiments.

Nur77 was reported to be implicated in inflammation and immunity, and its expression was significantly reduced in colon tissues in colitis [14]. Meanwhile, Nur77 participates in inducing autophagy to exert anti-inflammatory [21]. Interestingly, we identified that GPA induced Nur77 translocation from the nucleus to mitochondria by western blot and immunofluorescence (Figures 6(a)–6(d) and Figure S5A). It implied that the mechanism of GPA mediating autophagy may be related to Nur77. Then, we used a coimmunoprecipitation (CoIP) assay with endogenous Nur77 protein, which showed that GPA could enhance the interaction of Nur77 with TRAF6 and p62 (Figures 6(e) and 6(f)). The binding was confirmed by endogenous TRAF6 protein, which showed that GPA promoted Nur77 interaction with TRAF6 (Figure S5B).

To further confirm whether GPA induced autophagy via Nur77, TRAF6, and p62, Nur77, TRAF6, and p62 were knocked down in MODE-K cells; the western blot results showed that autophagy activation by GPA was abolished after Nur77, TRAF6, and p62 were knocked down (Figures 6(g) and 6(h) and Fig. S5CD). Overall, our findings indicated that GPA promoted Nur77 to colocalization at mitochondria, resulting in enhancing Nur77 interaction with TRAF6 and p62 to induce autophagy.

### 3.7. Nur77 Mediates the Anti-Inflammatory and Antioxidant Effects of GPA in IECs

**Figure 7:**
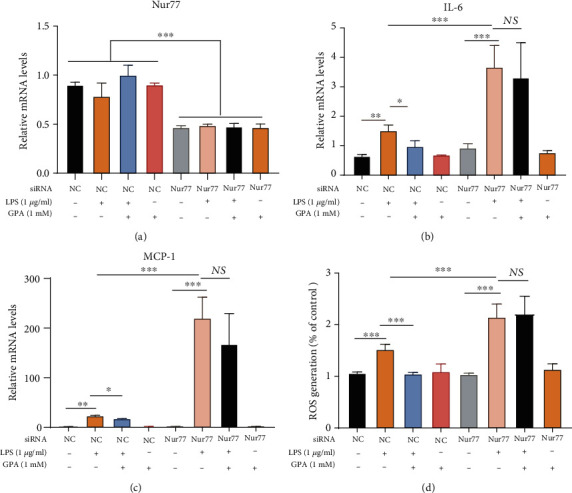
Nur77 mediates the anti-inflammatory and antioxidant effects of GPA in IECs. MODE-K cells were transfected with control or Nur77siRNA and further stimulated with GPA (1 mM) for 6 h, followed by LPS (1 *μ*g/ml) for 3 h; levels of Nur77, IL-6, and MCP-1 were analyzed by qPCR (a, b, c). The intracellular ROS levels were measured by DCF fluorescence microscopy (d). The results are expressed as the mean ± SD, three independent experiments. ^∗^*p* < 0.05, ^∗∗^*p* < 0.01, and ^∗∗∗^*p* < 0.001 vs. the control group; ^#^*p* < 0.05, ^##^*p* < 0.01, and ^###^*p* < 0.001 vs. the LPS-treated group.

Subsequently, we evaluated whether the anti-inflammatory and antioxidant effects of GPA were depended on Nur77; Nur77 were knocked down in MODE-K cells (Figure 7(a)). We found that knockdown Nur77 aggravated IL-6, MCP-1, and ROS production, induced by LPS (Figures 7(b)–7(d)). And the inhibitory effects of GPA on IL-6, MCP-1, and ROS production were blocked after Nur77 was knockdown (Figures 7(b)–7(d)). And GPA alone treatment has no effect on proinflammatory cytokines and ROS (Figures 7(a)–7(d)). It indicated that GPA could exert anti-inflammatory and antioxidant effects through Nur77.

## 4. Discussion

IBD causes digestive tract distress, weight loss, and gastrointestinal permeability affecting the health of millions of people worldwide [2, 4, 45, 46]. The morbidity of IBD is on the rise; however, current IBD therapeutic agents such as salicylazosulfapyridine (SASP), immunosuppressive agents, and anti-TNF-*α* monoclonal antibodies have showed limited efficacy and potential long-term toxicity [47, 48]. Hence, it is important to apply an external source of anti-inflammatory agents to cope with the disease. In our study, we found GPA peptide, without affecting cell viability, could alleviate DSS-induced colitis.

Overproduction of proinflammatory cytokines and oxidative stress is a hallmark of colon damage in the development of IBD [49–52]. Proinflammatory cytokines, i.e., IL-6 and TNF-*α*, would recruit and activate immune cells, including regulatory T cells and helper T cells [53]. And in the gut, ROS as a signal molecule, is majorly produced in mitochondrial, would stimulate inflammatory signal and plays a central role in colitis [54]. Overproduction of proinflammatory cytokine and ROS would amplify immune signal and exacerbate the breakdown of the epithelium [8, 9, 55]. Hence, controlling inflammation and oxidative is an effective way to treat IBD in clinical trials [8, 56]. In our study, GPA successfully alleviates acute inflammation by suppressing the high production of proinflammatory cytokines, ROS, and MDA in vivo and in vitro. Moreover, GPA also benefits the intestinal epithelial barrier, through maintaining tight junction. These results suggest that GPA would inhibit inflammatory response and oxidative stress, and simultaneously repair the epithelial barrier, which may provide a dual-effective agent for the remission of colitis.

Nur77 plays a center role in immune inflammatory response and was found to be implicated in several inflammatory diseases, such as diabetes, sepsis, pulmonary arterial hypertension, and IBD [13, 14, 57]. In IBD, the level of Nur77 is significantly reduced in colon tissues, and loss of Nur77 aggravates the development of colitis [14]. Nur77 mainly resides in the nucleus, but upon stimulation, it can be translocated to the mitochondria to trigger autophagy and apoptosis [58–60]. In HepG2 cells, it was demonstrated that celastrol promotes Nur77 ubiquitination and induces its translocation to mitochondria, resulting in induction of autophagy and suppression of inflammation [21]. Meanwhile, autophagy represents an effective way to control inflammatory responses and oxidative stress [42, 61–66]. In our study, we found that GPA treatment promotes Nur77 translocation from the nucleus to mitochondria, then enhancing Nur77 interaction with TRAF6 and p62 to induce autophagy, resulting in suppression inflammatory and oxidative stress. Our current studies revealed that inhibition of autophagy, through chlorquinaldol, activates NF-*κ*B, and activation of autophagy by rapamycin suppresses NF-*κ*B in IECs, revealing that chlorquinaldol activated NF-*κ*B through suppressing the fusion stage in IECs. Rapamycin activates autophagy by targeting mTOR, and chlorquinadol inhibits autophagy by targeting autolysosome, and these were common activator and inhibitor of autophagy [67, 68]. To the best of our knowledge, this is the first report that Nur77 is involved in the regulation of autophagy to suppress inflammatory response and oxidative stress of intestinal epithelial cells.

In conclusion, our work explores a novel therapeutic strategy for IBD. GPA significantly attenuates DSS-induced colitis in vivo and in vitro. GPA treatment recruites Nur77 from the nucleus to mitochondria, promoting Nur77 interaction with TRAF6 and p62, leading to autophagy activation, and suppressing inflammation and oxidative stress. Our results suggested that GPA could potentially be used for the treatment of IBD.

## Data Availability

The data used to support the findings of this study are available from the corresponding author upon request.

## Abbreviations

IBD: Inflammatory bowel disease
CD: Crohn's disease
UC: Ulcerative colitis
IECs: Intestinal epithelial cells
GPA: Gly-Pro-Ala
DSS: Dextra sulfate sodium
LPS: Lipopolysaccharides
TNF-*α*: Tumor necrosis factor-*α*
IL-6: Interleukin 6
MCP-1: Monocyte chemoattractant protein-1
TRAF6: Tumor necrosis factor receptor-associated factor 6
DAI: Disease activity index
MDA: Malondialdehyde.

## Supplementary Materials

Supplementary Materials Figure S1: GPA inhibits DSS-induced colitis in vivo. Figure S2: GPA inhibits inflammation induced by LPS. Figure S3: GPA inhibits cytotoxicity and inflammation induced by TNF-*α*. Figure S4: GPA induces autophagy. Figure S5: GPA promotes Nur77 interaction with TRAF6 and p62 and induces autophagy. Table S1: primers used for RT-PCR analysis. Table S2: primers used for siRNA analysis.

## References

[1] Xavier R. J., Podolsky D. K. (2007). Unravelling the pathogenesis of inflammatory bowel disease. *Nature*.

[2] Ordás I., Eckmann L., Talamini M., Baumgart D. C., Sandborn W. J. (2012). Ulcerative colitis. *Lancet*.

[3] Olivera P., Danese S., Jay N., Natoli G., Peyrin-Biroulet L. (2019). Big data in IBD: a look into the future. *Nature Reviews Gastroenterology & Hepatology*.

[4] Cosnes J., Gower–Rousseau C., Seksik P., Cortot A. (2011). Epidemiology and natural history of inflammatory bowel diseases. *Gastroenterology*.

[5] Bouma G., Strober W. (2003). The immunological and genetic basis of inflammatory bowel disease. *Nature Reviews Immunology*.

[6] Macdonald T. T., Monteleone G. (2005). Immunity, inflammation, and allergy in the gut. *Science*.

[7] Korzenik J. R., Podolsky D. K. (2006). Evolving knowledge and therapy of inflammatory bowel disease. *Nature Reviews Drug Discovery*.

[8] Eisenstein M. (2018). Gut reaction. *Nature*.

[9] Plichta D. R., Graham D. B., Subramanian S., Xavier R. J. (2019). Therapeutic opportunities in inflammatory bowel disease: mechanistic dissection of host-microbiome relationships. *Cell*.

[10] Zhang M., Sun K., Wu Y., Yang Y., Tso P., Wu Z. (2017). Interactions between intestinal microbiota and host immune response in inflammatory bowel disease. *Frontiers in Immunology*.

[11] Li L., Liu Y., Chen H. Z. (2015). Impeding the interaction between Nur77 and p38 reduces LPS-induced inflammation. *Nature Chemical Biology*.

[12] Banno A., Lakshmi S. P., Reddy A. T., Kim S. C., Reddy R. C. (2019). Key functions and therapeutic prospects of Nur77 in inflammation related lung diseases. *The American Journal of Pathology*.

[13] Kurakula K., Sun X. Q., Happé C. (2019). 6-Mercaptopurine, an agonist of Nur77, reduces progression of pulmonary hypertension by enhancing BMP signalling. *The European Respiratory Journal*.

[14] Wu H., Li X. M., Wang J. R. (2016). NUR77 exerts a protective effect against inflammatory bowel disease by negatively regulating the TRAF6/TLR–IL-1R signalling axis. *Journal of Pathology*.

[15] Wang J. R., Gan W. J., Li X. M. (2014). Orphan nuclear receptor Nur77 promotes colorectal cancer invasion and metastasis by regulating MMP-9 and E-cadherin. *Carcinogenesis*.

[16] Kanzleiter T., Preston E., Wilks D. (2010). Overexpression of the orphan receptor Nur77 alters glucose metabolism in rat muscle cells and rat muscle in vivo. *Diabetologia*.

[17] De Silva S., Han S., Zhang X., Huston D. P., Winoto A., Zheng B. (2005). Reduction of the incidence and severity of collagen-induced arthritis by constitutive Nur77 expression in the T cell lineage. *Arthritis and Rheumatism*.

[18] Rath E., Moschetta A., Haller D. (2018). Mitochondrial function - gatekeeper of intestinal epithelial cell homeostasis. *Nature Reviews Gastroenterology & Hepatology*.

[19] Mottawea W., Chiang C. K., Mühlbauer M. (2016). Altered intestinal microbiota–host mitochondria crosstalk in new onset Crohn’s disease. *Nature Communications*.

[20] Yin H., Zhang S., Sun Y. (2017). MicroRNA-34/449 targets IGFBP-3 and attenuates airway remodeling by suppressing Nur77-mediated autophagy. *Cell Death & Disease*.

[21] Hu M., Luo Q., Alitongbieke G. (2017). Celastrol-induced Nur77 interaction with TRAF2 alleviates inflammation by promoting mitochondrial ubiquitination and autophagy. *Molecular Cell*.

[22] Chalamaiah M., Yu W., Wu J. (2018). Immunomodulatory and anticancer protein hydrolysates (peptides) from food proteins: a review. *Food Chemistry*.

[23] Martínez-Alvarez O., Chamorro S., Brenes A. (2015). Protein hydrolysates from animal processing by-products as a source of bioactive molecules with interest in animal feeding: a review ☆. *Food Research International*.

[24] Zheng L., Wei H., Yu H. (2018). Fish skin gelatin hydrolysate production by ginger powder induces glutathione synthesis to prevent hydrogen peroxide induced intestinal oxidative stress via the Pept1-p62-Nrf2 cascade. *Journal of Agricultural and Food Chemistry*.

[25] Zheng L., Yu H., Wei H. (2018). Antioxidative peptides of hydrolysate prepared from fish skin gelatin using ginger protease activate antioxidant response element-mediated gene transcription in IPEC-J2 cells. *Journal of Functional Foods*.

[26] Vidal K., Grosjean I., Revillard J. P., Gespach C., Kaiserlian D. (1993). Immortalization of mouse intestinal epithelial cells by the SV40-large T gene : phenotypic and immune characterization of the MODE-K cell line. *Journal of Immunological Methods*.

[27] Crockett E. K., Washburn K. F., Graef J. L., Lucas E. A., Smith B. J. (2017). MODE-K cells as a model to study the gut epithelial response: an outside-in and inside-out approach. *The FASEB Journal*.

[28] Zhang H., Kovacs-Nolan J., Kodera T., Eto Y., Mine Y. (2015). *γ*-Glutamyl cysteine and *γ*-glutamyl valine inhibit TNF-*α* signaling in intestinal epithelial cells and reduce inflammation in a mouse model of colitis via allosteric activation of the calcium-sensing receptor. *Biochimica et Biophysica Acta (BBA)-Molecular Basis of Disease*.

[29] Kovacs-Nolan J., Zhang H., Ibuki M. (2012). The PepT1-transportable soy tripeptide VPY reduces intestinal inflammation. *Biochimica et Biophysica Acta (BBA)-General Subjects*.

[30] Dieleman L. A., Palmen M. J., Akol H. (1998). Chronic experimental colitis induced by dextran sulphate sodium (DSS) is characterized by Th1 and Th2 cytokines. *Clinical and Experimental Immunology*.

[31] Deng Z., Cui C., Wang Y. (2020). FSGHF3 and peptides, prepared from fish skin gelatin, exert a protective effect on DSS-induced colitis via the Nrf2 pathway. *Food & Function*.

[32] Huang Y., Jiang H., Chen Y. (2018). Tranilast directly targets NLRP3 to treat inflammasome-driven diseases. *EMBO Molecular Medicine*.

[33] Zilu S., Qian H., Haibin W. (2019). Effects of XIAP on high fat diet-induced hepatic steatosis: a mechanism involving NLRP3 inflammasome and oxidative stress. *Aging*.

[34] Wang R., Luo Y., Lu Y. (2019). Maggot extracts alleviate inflammation and oxidative stress in acute experimental colitis via the activation of Nrf2. *Oxidative Medicine and Cellular Longevity*.

[35] Rezaie A., Parker R. D., Abdollahi M. (2007). Oxidative stress and pathogenesis of inflammatory bowel disease: an epiphenomenon or the cause?. *Digestive Diseases and Sciences*.

[36] Aviello G., Knaus U. G. (2018). NADPH oxidases and ROS signaling in the gastrointestinal tract. *Mucosal Immunology*.

[37] Snezhkina A. V., Kudryavtseva A. V., Kardymon O. L. (2019). ROS generation and antioxidant defense systems in normal and malignant cells. *Oxidative Medicine and Cellular Longevity*.

[38] McGuckin M. A., Eri R., Simms L. A., Florin T. H. J., Radford-Smith G. (2009). Intestinal barrier dysfunction in inflammatory bowel diseases. *Inflammatory Bowel Diseases*.

[39] Bashashati M., Habibi H. R., Keshavarzian A., Schmulson M., Sharkey K. A. (2015). Intestinal microbiota: a regulator of intestinal inflammation and cardiac ischemia?. *Current Drug Targets*.

[40] Hwang J. R., Oh S. Y., Choi S. J., Kim J. S., Kim J. H., Roh C. R. (2014). Inhibition of autophagy enhances NF-kB activity in JEG-3 cells. *Placenta*.

[41] Levine B., Mizushima N., Virgin H. W. (2011). Autophagy in immunity and inflammation. *Nature*.

[42] Zhong Z., Sanchez-Lopez E., Karin M. (2016). Autophagy, inflammation, and immunity: a troika governing cancer and its treatment. *Cell*.

[43] Vikram A., Anish R., Kumar A., Tripathi D. N., Kaundal R. K. (2017). Oxidative stress and autophagy in metabolism and longevity. *Oxidative Medicine and Cellular Longevity*.

[44] Cordani M., Donadelli M., Strippoli R., Bazhin A. V., Sanchez-Alvarez M. (2019). Interplay between ROS and autophagy in cancer and aging: from molecular mechanisms to novel therapeutic approaches. *Oxidative Medicine and Cellular Longevity*.

[45] Welcker K., Martin A., Kölle P., Siebeck M., Gross M. (2004). Increased intestinal permeability in patients with inflammatory bowel disease. *European Journal of Medical Research*.

[46] Wang L., Tang H., Wang C., Hu Y., Wang S., Shen L. (2019). Aquaporin 4 deficiency alleviates experimental colitis in mice. *The FASEB Journal*.

[47] Bressler B., Marshall J. K., Bernstein C. N. (2015). Clinical practice guidelines for the medical management of nonhospitalized ulcerative colitis: the Toronto consensus. *Gastroenterology*.

[48] Moura F. A., de Andrade K. Q., dos Santos J. C. F., Araújo O. R. P., Goulart M. O. F. (2015). Antioxidant therapy for treatment of inflammatory bowel disease: does it work?. *Redox Biology*.

[49] Chen Z. J. (2012). Ubiquitination in signaling to and activation of IKK. *Immunological Reviews*.

[50] Hayden M. S., Ghosh S. (2008). Shared principles in NF-kappaB signaling. *Cell*.

[51] Karin M., Gallagher E. (2009). TNFR signaling: ubiquitin-conjugated TRAFfic signals control stop-and-go for MAPK signaling complexes. *Immunological Reviews*.

[52] Serra G., Incani A., Serreli G. (2018). Olive oil polyphenols reduce oxysterols-induced redox imbalance and pro-inflammatory response in intestinal cells. *Redox Biology*.

[53] Friedrich M., Pohin M., Powrie F. (2019). Cytokine networks in the pathophysiology of inflammatory bowel disease. *Immunity*.

[54] Novak E. A., Mollen K. P. (2015). Mitochondrial dysfunction in inflammatory bowel disease. *Frontiers in cell and developmental biology*.

[55] Kumar S., Suman S., Fornace A. J., Datta K. (2019). Intestinal stem cells acquire premature senescence and senescence associated secretory phenotype concurrent with persistent DNA damage after heavy ion radiation in mice. *Aging*.

[56] Martin J. C., Chang C., Boschetti G. (2019). Single-cell analysis of Crohn’s disease lesions identifies a pathogenic cellular module associated with resistance to anti-TNF therapy. *Cell*.

[57] Palumbo-Zerr K., Zerr P., Distler A. (2015). Orphan nuclear receptor NR4A1 regulates transforming growth factor-*β* signaling and fibrosis. *Nature Medicine*.

[58] Zhang X.-k. (2006). Targeting Nur77 translocation. *Expert Opinion on Therapeutic Targets*.

[59] Zhan Y., du X., Chen H. (2008). Cytosporone B is an agonist for nuclear orphan receptor Nur77. *Nature Chemical Biology*.

[60] Cheng Z., Völkers M., Din S. (2011). Mitochondrial translocation of Nur77 mediates cardiomyocyte apoptosis. *European Heart Journal*.

[61] Nunnari J., Suomalainen A. (2012). Mitochondria: in sickness and in health. *Cell*.

[62] Green D. R., Galluzzi L., Kroemer G. (2011). Mitochondria and the autophagy–inflammation–cell death axis in organismal aging. *Science*.

[63] Gurung P., Lukens J. R., Kanneganti T. D. (2015). Mitochondria: diversity in the regulation of the NLRP3 inflammasome. *Trends in Molecular Medicine*.

[64] Iida T., Yokoyama Y., Wagatsuma K., Hirayama D., Nakase H. (2019). Impact of autophagy of innate immune cells on inflammatory bowel disease. *Cell*.

[65] Kimura T., Isaka Y., Yoshimori T. (2017). Autophagy and kidney inflammation. *Autophagy*.

[66] Lu R., Zhang Y. G., Xia Y., Sun J. (2019). Imbalance of autophagy and apoptosis in intestinal epithelium lacking the vitamin D receptor. *The FASEB Journal*.

[67] Rangaraju S., Verrier J. D., Madorsky I., Nicks J., Dunn W. A., Notterpek L. (2010). Rapamycin activates autophagy and improves myelination in explant cultures from neuropathic mice. *The Journal of Neuroscience*.

[68] Kaur J., Debnath J. (2015). Autophagy at the crossroads of catabolism and anabolism. *Nature Reviews. Molecular Cell Biology*.

