# Supplementary material for: GPA Peptide-Induced Nur77 Localization at Mitochondria Inhibits Inflammation and Oxidative Stress through Activating Autophagy in the Intestine
Source: Oxid Med Cell Longev. 2020 Aug 20;2020:4964202. doi: 10.1155/2020/4964202 (PMC7456482; doi:10.1155/2020/4964202)
Supplement: Supplementary Materials — Figure S1: GPA inhibits DSS-induced colitis in vivo. Figure S2: GPA inhibits inflammation induced by LPS. Figure S3: GPA inhibits cytotoxicity and inflammation induced by TNF-α. Figure S4: GPA induces autophagy. Figure S5: GPA promotes Nur77 interaction with TRAF6 and p62 and induces autophagy. Table S1: primers used for RT-PCR analysis. Table S2: primers used for siRNA analysis. [file 4964202.f1.pdf]

Supplementary material

1. Supplementary figures

2. Supplementary tables

Figure legends

Figure S1 GPA inhibits DSS-induced colitis in vivo

Weight of mice during the experiment (A). Disease activity index (DAI) during the disease process (B). The lengths of colons from each group of mice were measured (C). *p < 0.05 vs the control group; #p < 0.05 vs the DSS-treated group, (n = 6/group).

Figure S2 GPA inhibits inflammation induced by LPS

MODE-K cells were treated with different GPA concentrations for 24 h, and the cell viability was detected by MTT assay (A). MODE-K cells pre-treated with different concentrations of GPA for 6 h were exposed to LPS (1 μg/ml) for 3 h. MCP-1, IL-6 and TNFα mRNA levels were detected by quantitative real-time PCR (B). The results are expressed as mean ± SD, three independent experiments. *p < 0.05, **p < 0.01 and ***p < 0.001 vs the control group; #p < 0.05, ##p < 0.01 and ###p < 0.001 vs the LPS-treated group.

Figure S3 GPA inhibits cytotoxicity and inflammation induced by TNF-α

MODE-K cells pre-treated with different concentrations of GPA for 6 h were exposed to TNF-α (10 ng/ml) for 3 h. The cytotoxicity was detected by LDH assay (A). MCP-1 and IL-6 mRNA levels were detected by quantitative real-time PCR (B). The results are expressed as mean ± SD, three independent experiments. *p < 0.05, **p < 0.01 and ***p < 0.001 vs the control group; #p < 0.05, ##p < 0.01 and ###p < 0.001 vs the LPS-treated group.

Figure S4 GPA induces autophagy

LC3 levels were analyzed by immunofluorescence in colon tissues of mice. Nur77

(green) and DAPI (blue). Scale bar, 5 μm (A). MODE-K cells pre-treated with different concentrations of GPA for 6 h were exposed to TNF-α (10 ng/ml) for 6 h, the protein level of LC3 was analyzed by western blot (B). MODE-K cells pre-treated with different concentrations of GPA for 6 h were exposed to LPS (1 μg/ml) for 3 h, mitochondria were separated by Cell Mitochondria Isolation Kit, and the level of LC3 in the mitochondria was detected by western blot (C).

Figure S5 GPA promotes Nur77 interaction with TRAF6 and p62 and induces autophagy.

MODE-K cells were stimulated with GPA (1 mM) for 6 h, followed by LPS (1 μg/mL) for 3 h. Mitochondria were separated by Cell Mitochondria Isolation Kit, and the level of COX4, β-tubulin, PCNA in the mitochondria, cell nucleus and cytoplasm were detected by western blot (A). IP and immunoblot analysis of the interaction of endogenous TRAF6 with Nur77 (B). MODE-K cells were transfected with control or p62 - siRNA or TRAF6 - siRNA, MODE-K cells were stimulated with GPA (1 mM) for 6 h, followed by LPS (1 μg/mL) for 3 h, levels of p62, TRAF6 and LC3 in cell lysate were analyzed by western blot (CD). Three independent experiments.

Table S1. Primers used for RT-PCR analysis

Table S2. Primers used for siRNA analysis

Fig. S1

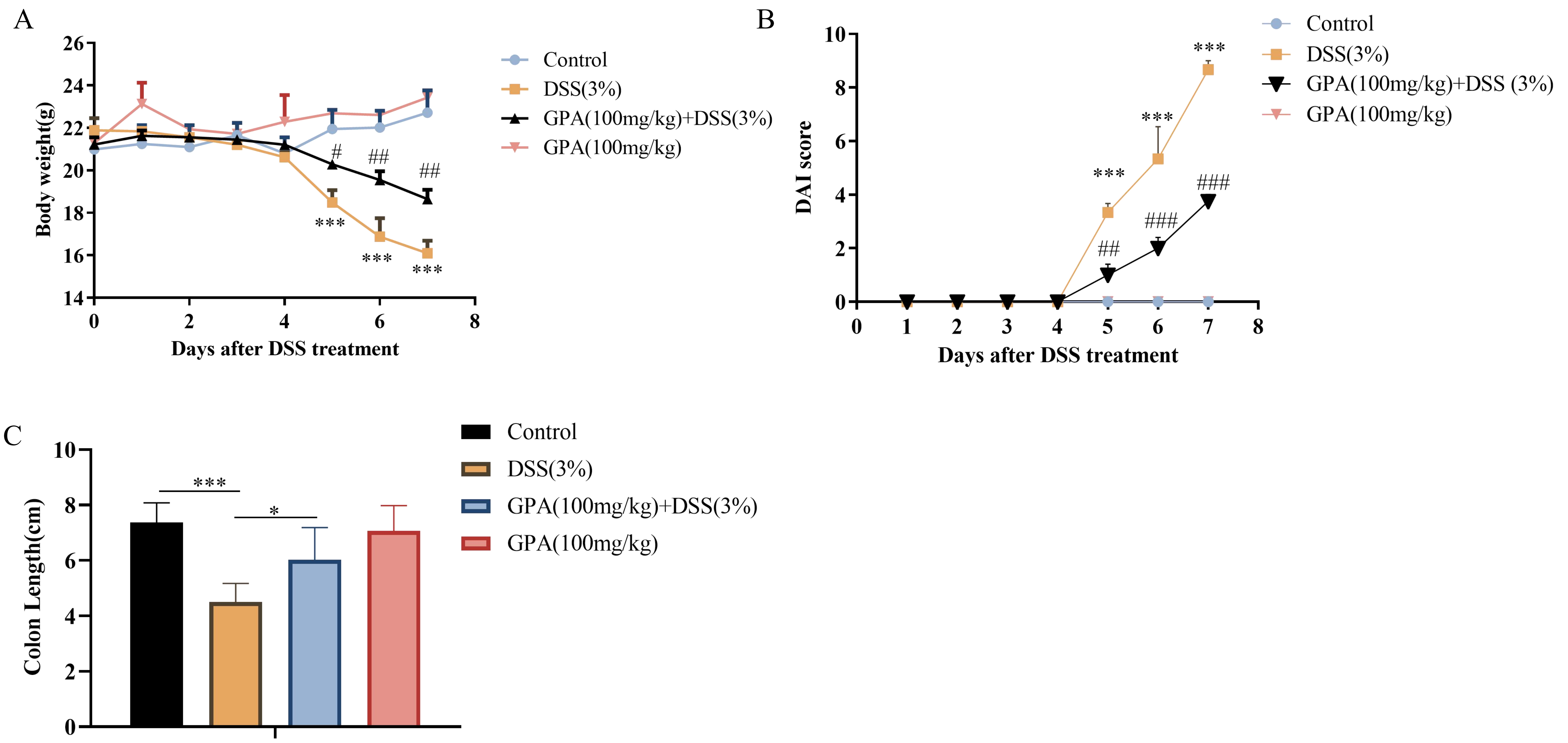
A
Body weight(g)
26
24
22
20
18
16
14
0
2
4
6
8
Days after DSS treatment
Control
DSS(3%)
GPA(100mg/kg)+DSS(3%)
GPA(100mg/kg)
#
##
##
***
***
***
B
DAI score
10
8
6
4
2
0
0
1
2
3
4
5
6
7
8
Days after DSS treatment
Control
DSS(3%)
GPA(100mg/kg)+DSS (3%)
GPA(100mg/kg)
***
***
***
###
###
##
C
Colon Length(cm)
10
8
6
4
2
0
***
*
Control
DSS(3%)
GPA(100mg/kg)+DSS(3%)
GPA(100mg/kg)

Fig. S2

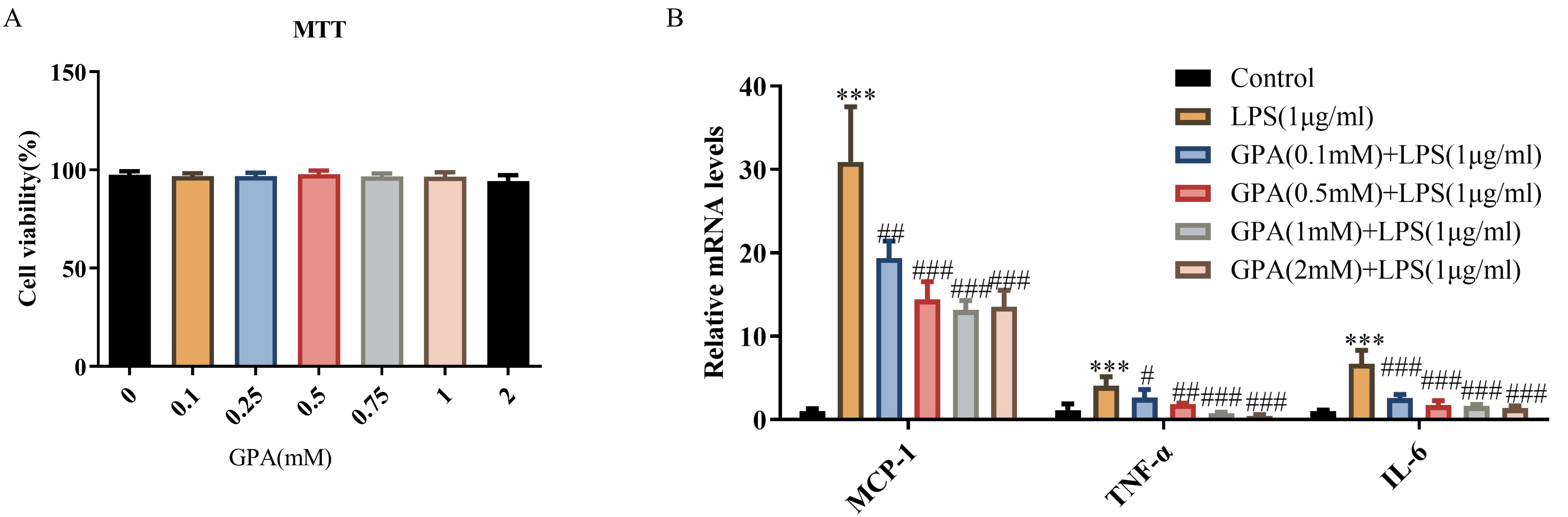

A
MTT
Cell viability(%)
150
100
50
0
0
0.1
0.25
0.5
0.75
1
2
GPA(mM)
B
Relative mRNA levels
40
30
20
10
0
***
##
###
###
###
***
#
##
###
###
***
###
###
###
###
MCP-1
TNF-α
IL-6
Control
LPS(1μg/ml)
GPA(0.1mM)+LPS(1μg/ml)
GPA(0.5mM)+LPS(1μg/ml)
GPA(1mM)+LPS(1μg/ml)
GPA(2mM)+LPS(1μg/ml)

Fig. S3

A

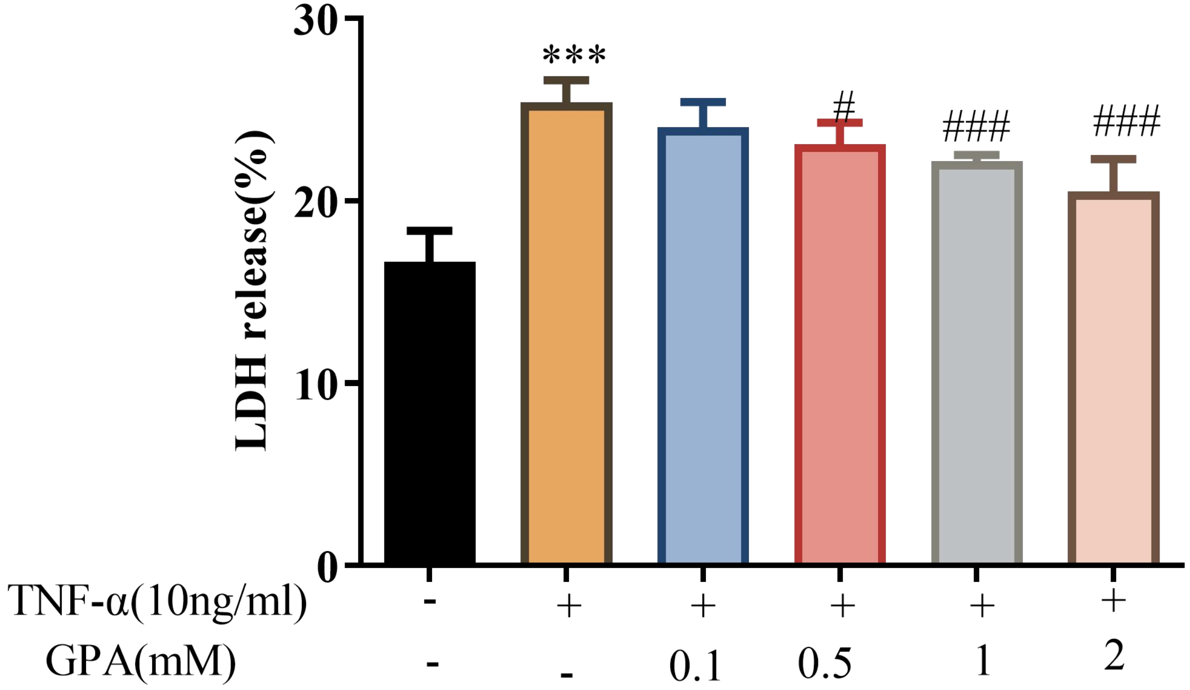

30
20
10
0
LDH release(%)
***
#
###
###
TNF-α(10ng/ml) - + + + + +
GPA(mM) - - 0.1 0.5 1 2

B

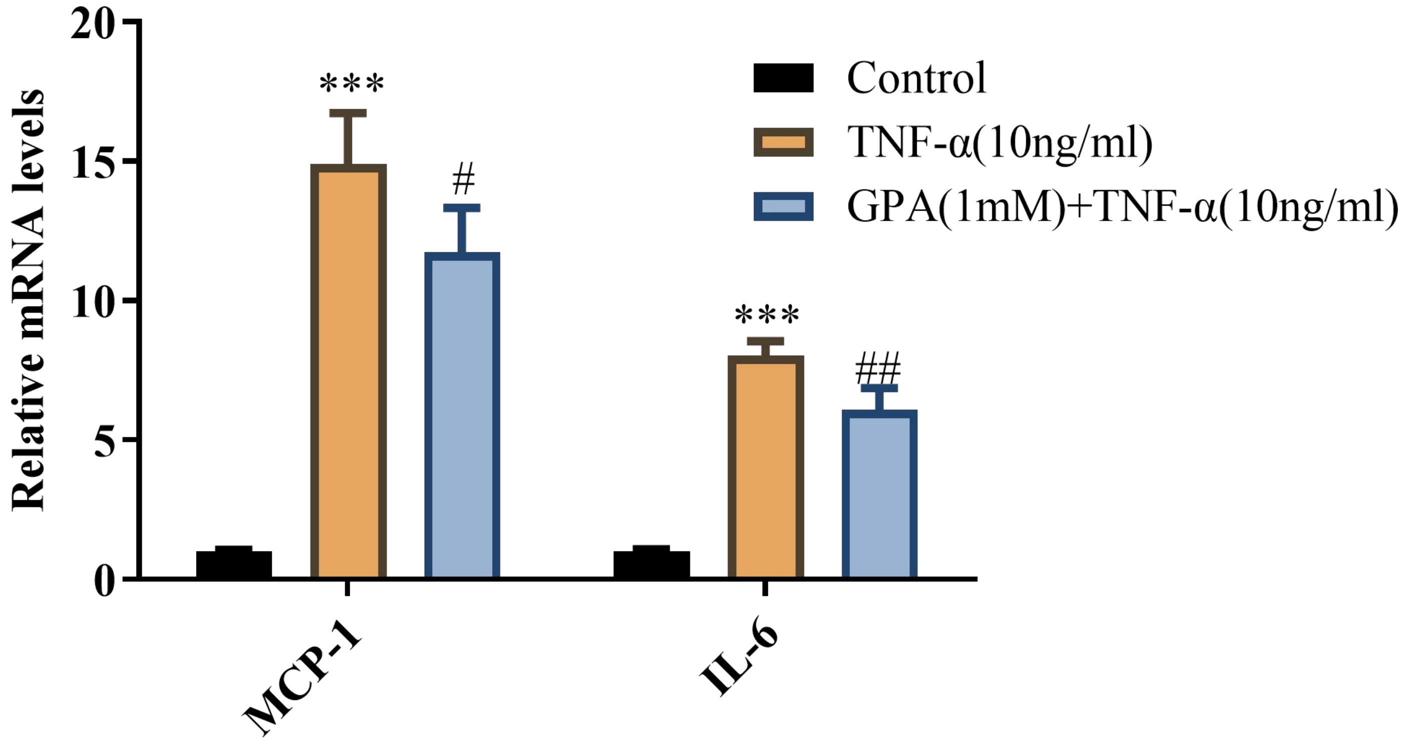

20
15
10
5
0
Relative mRNA levels
***
#
***
##
Control
TNF-α(10ng/ml)
GPA(1mM)+TNF-α(10ng/ml)
MCP-1
IL-6

Fig. S4

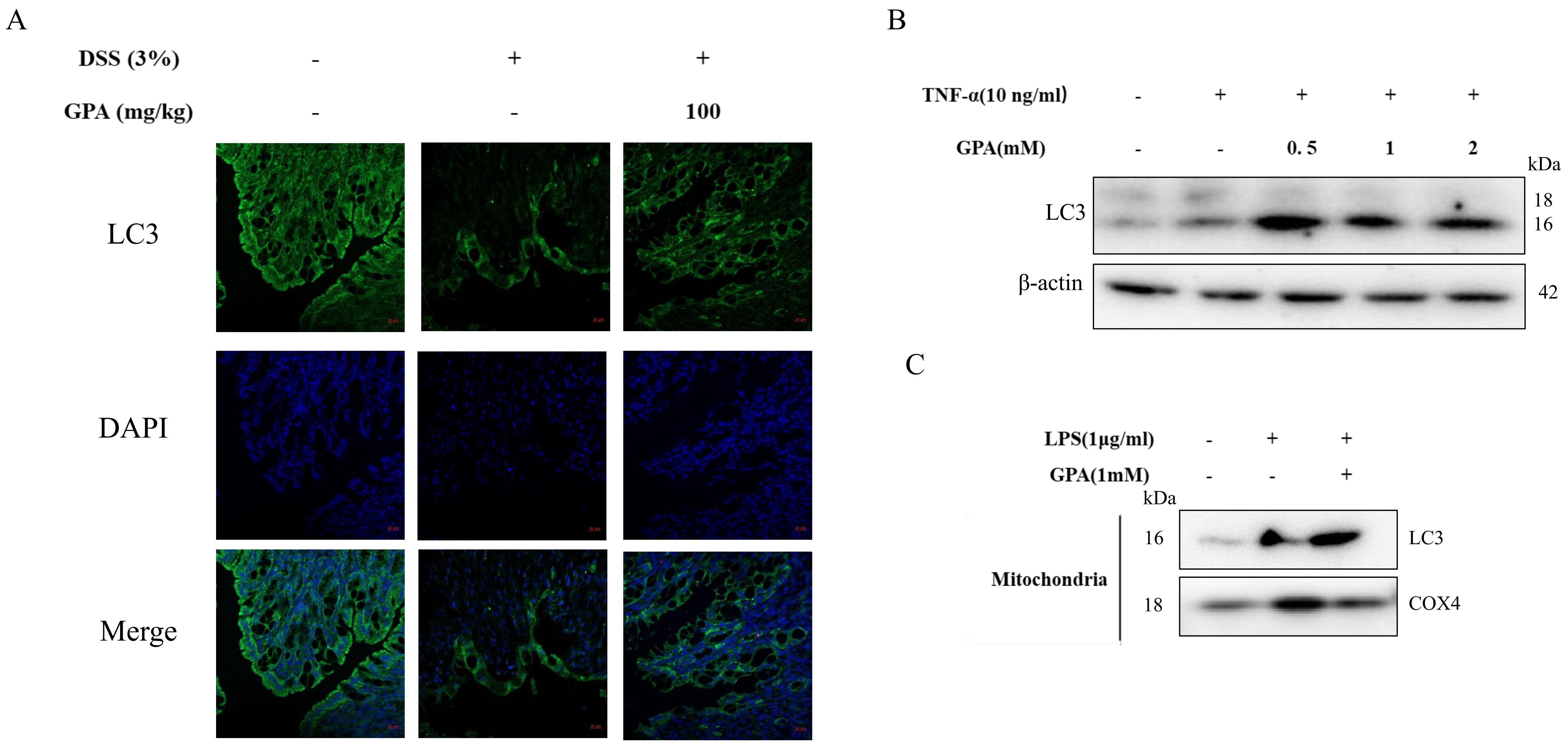
A
DSS (3%)
-
+
+
GPA (mg/kg)
-
-
100
LC3
DAPI
Merge
B
TNF-α(10 ng/ml)
-
+
+
+
+
GPA(mM)
-
-
0. 5
1
2
kDa
LC3
18
16
β-actin
42
C
LPS(1μg/ml)
-
+
+
GPA(1mM)
-
-
+
kDa
Mitochondria
16
LC3
18
COX4

Fig. S5

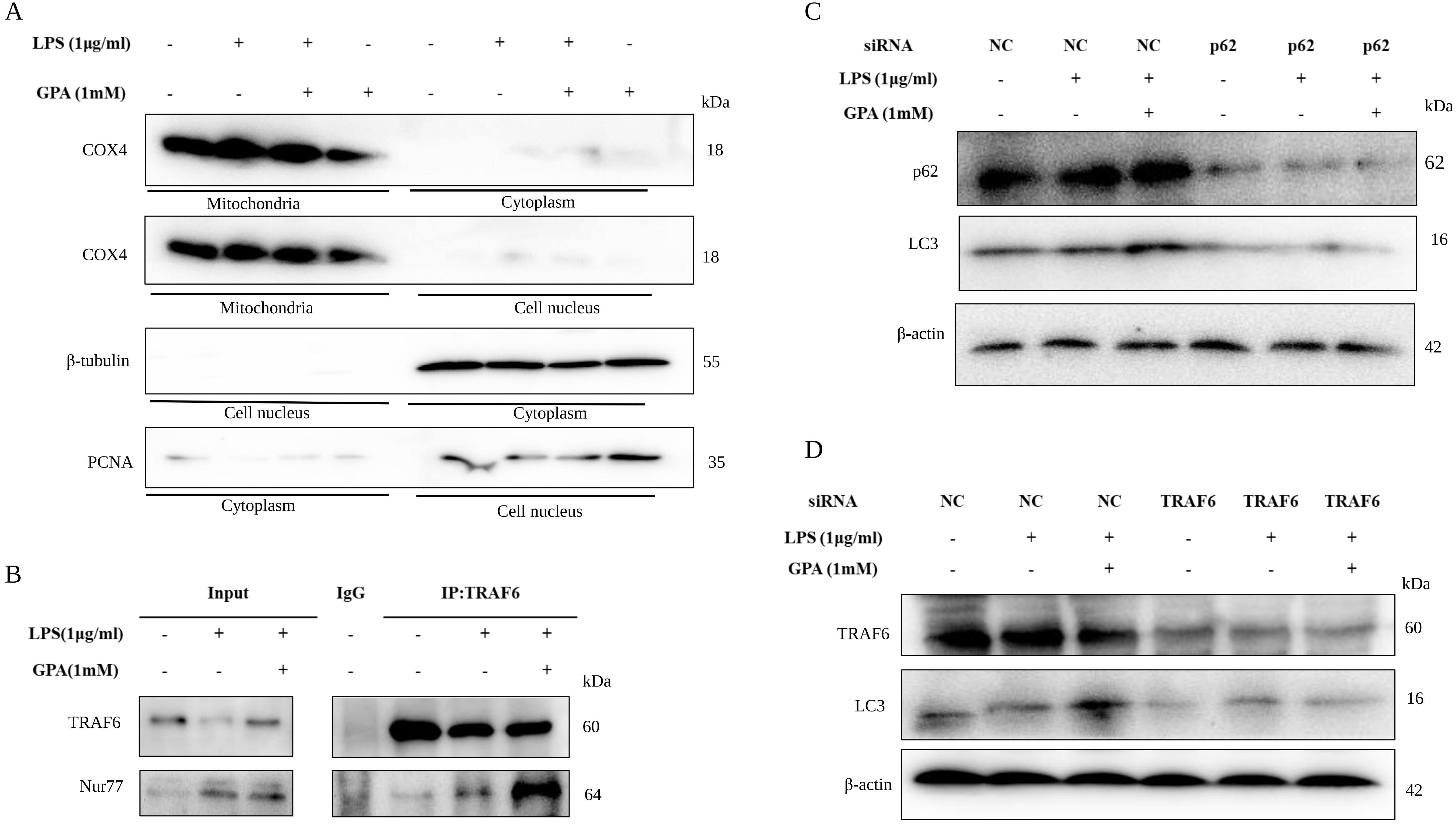
A
LPS (1μg/ml) - + + - - + + -
GPA (1mM) - - + + - - + +
kDa
COX4 18
Mitochondria
Cytoplasm
COX4 18
Mitochondria
Cell nucleus
β-tubulin 55
Cell nucleus
Cytoplasm
PCNA 35
Cytoplasm
Cell nucleus
B
Input
IgG
IP:TRAF6
LPS(1μg/ml) - + + - - + +
GPA(1mM) - - + - - - +
kDa
TRAF6 60
Nur77 64
C
siRNA NC NC NC p62 p62 p62
LPS (1μg/ml) - + + - + +
GPA (1mM) - - + - - +
kDa
p62 62
LC3 16
β-actin 42
D
siRNA NC NC NC TRAF6 TRAF6 TRAF6
LPS (1μg/ml) - + + - + +
GPA (1mM) - - + - - +
kDa
TRAF6 60
LC3 16
β-actin 42

Table S1. Primers used for RT-PCR analysis

Primer	Primer sequences (5′ → 3′)	Product size (bp)
m-β-actin	GGCACCACACCTTCTACAATG<br>GGGGTGTTGAAGGTCTCAAAC	133
m-GAPDH	AACTTTGGCATTGTGGAAGG<br>GGATGCAGGGATGATGTTCT	132
m-MCP-1	AGCACCAGCCAACTCTCAC<br>TCTGGACCCATTCCTTCTTG	301
m-IL-6	CCGGAGAGGAGACTTCACAG<br>CAGAATTGCCATTGCACAAC	134
m-TNFα	CGGAGTCCGGGCAGGTCTACTTT<br>GTCCAGGTCACTGTCCCAGCATC	240
m-Nur77	ATGCTTCGTGTCAGCACTAT<br>GTACTTGGCGCTTTTCTGTA	86

Table S2. Primers used for siRNA analysis

Primer	sense (5′ → 3′)	antisense (5′ → 3′)
m-Nur77	CGGCGUCCUUCAAGUUUGATT	UCAAACUUGAAGGACGCCGTT
m-P62	GCUGAAACAUGGACACUUUTT	AAAGUGUCCAUGUUUCAGCTT
m-TRAF6	GCCAGGUUAAUACACACAUTT	AUGUGUGUAUUAACCUGGCTT
